# Curvature-Induced Spatial Ordering of Composition in Lipid Membranes

**DOI:** 10.1155/2017/7275131

**Published:** 2017-04-04

**Authors:** Shimrit Katz, Sefi Givli

**Affiliations:** Faculty of Mechanical Engineering, Technion–Israel Institute of Technology, 32000 Haifa, Israel

## Abstract

Phase segregation of membranal components, such as proteins, lipids, and cholesterols, leads to the formation of aggregates or domains that are rich in specific constituents. This process is important in the interaction of the cell with its surroundings and in determining the cell's behavior and fate. Motivated by published experiments on curvature-modulated phase separation in lipid membranes, we formulate a mathematical model aiming at studying the spatial ordering of composition in a two-component biomembrane that is subjected to a prescribed (imposed) geometry. Based on this model, we identified key nondimensional quantities that govern the biomembrane response and performed numerical simulations to quantitatively explore their influence. We reproduce published experimental observations and extend them to surfaces with geometric features (imposed geometry) and lipid phases beyond those used in the experiments. In addition, we demonstrate the possibility for curvature-modulated phase separation above the critical temperature and propose a systematic procedure to determine which mechanism, the difference in bending stiffness or difference in spontaneous curvatures of the two phases, dominates the coupling between shape and composition.

## 1. Introduction

The biological lipid bilayer membrane, or in short “biomembrane,” is a fundamental building block of the cell. It forms the barrier that separates the interior of the cell from its surroundings but is also responsible for almost all interaction of the cell with its environment, including transport, adhesion, regulation, transduction, and signaling [1–5]. The diverse functionality of the biomembrane is achieved by a seemingly simple structure, two layers that are primarily made from lipid molecules and also some integral proteins, cholesterols, and other functional molecules [6, 7]. This molecular structure of the biomembrane gives rise to the so-called “fluidity” of the membrane [8]; that is, its constituent molecules are able to move relatively easy within the membrane, which resists bending and stretching but not shear [9]. Consequently, biomembranes have a dynamic structure in the sense that their molecular arrangement (local composition) can change with conditions. For example, depending on temperature (and/or osmotic pressure, acidity, etc.) the biomembrane may possess a uniform mixture of its components or it may segregate into different phases, which are rich in specific constituent and possess different mechanical and chemical properties [10–16].

The fluidity of the biomembrane combined with its spatial heterogeneity brings about a unique coupling between shape (geometry) and composition. For example, lipid phases that possess high bending stiffness highly favor regions with small (magnitude) curvature [17]. Also, the three-dimensional molecular shape of some lipids and proteins results in a nonzero spontaneous curvature that affects the geometry of the biomembrane in their neighborhood. This two-way coupling between shape and composition means that deformations exhibited by biomembranes are strongly influenced by their heterogeneous composition, while the spatial ordering of composition is modulated by the geometry of the membrane [16, 18–23].

In the last two decades, much effort has been invested into understanding the consequences of the coupling between shape and composition in biomembranes. Theoretical models have generalized uniform composition models [24–27] to account for multicomponent or multiphase membranes [28–35]. Features such as equilibrium configurations, stability [36–39], interaction with the cytoskeleton [40–42], formation of lipid rafts, anisotropy of the membrane constituents [43], and even using biomembranes as sensors or actuators [44, 45] have been investigated. The complexity of the problem has forced the usage of sophisticated numerical methods, such as advanced phase field schemes, special nonlinear finite elements, and molecular dynamics simulations [17, 46–50], while analytical derivations have commonly adopted simplifying assumptions, like small deformations, axisymmetry, and so forth. The abovementioned theoretical studies have been motivated by a large body of experimental work, for example, [10–16, 21, 51–53], that demonstrated phenomena such as phase segregation, coexistence of different phases, and formation of domains in vesicles by a variety of methods, for example, fluorescence microscopy, X-ray diffraction, proton microscopy, spin resonance, and NMR imaging.

In a recent work, Parthasarathy et al. [54] designed an elegant experiment that breaks the two-way coupling between shape and composition and enables direct investigation of the influence of the membrane geometry on the spatial ordering of its composition. To this end, they used a quartz substrate, which was topographically patterned using photolithographic microfabrication techniques. The substrate consisted of continuously alternating high and low curvature contours with one-dimensional periodicity of 2 *μ*m. In order to decouple the main membrane from the underlying substrate a double membrane system was used: first, a supported membrane of uniform composition was deposited on the substrate. Then, the “main” membrane, a giant unilamellar vesicle (GUV), was introduced on top. Parthasarathy et al. showed that beyond a critical temperature, the spatial organization of lipid phases can be directed by gradients of membrane curvature, provided that these gradients are large enough.

In the current paper we analyze this type of experiment by means of a mathematical model combined with numerical simulations. The main goal is to reproduce the experimental observations mentioned above but also to generalize them and motivate new experiments. Accordingly, the structure of the paper is as follows: Section 2 describes the main theoretical considerations, governing equations, and nondimensional quantities that govern the spatial ordering of composition in a biomembrane subjected to imposed geometry. In Section 3, we perform a numerical study aiming at understanding the role of the nondimensional quantities that were identified in Section 2, and in particular their influence on the evolution of the spatial organization of the biomembrane composition. Focus is put on the final (steady state) spatial field of the membrane composition. The main conclusions are discussed in Section 4.

## 2. Theoretical Considerations

### 2.1. Governing Equations

**Figure 1:**
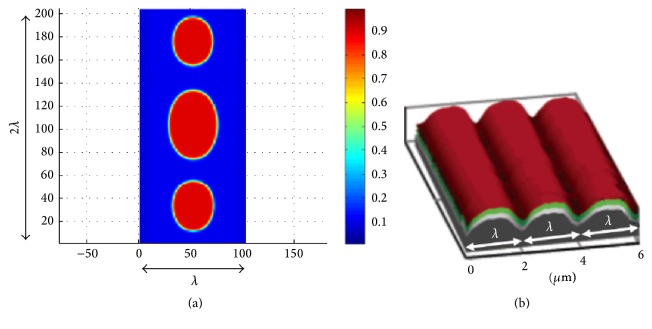
Composition field, *ϕ*, is calculated in a rectangular section of dimension *λ* × 2*λ*, typically discretized into 100 × 200 points, and subjected to periodic boundary conditions. Here, *λ* is the 1D period of the surface curvature in the experiment. The value of *ϕ* ∈ [0,1] at each point is shown by different colors (see color bar). The image on the right shows a schematic illustration of the topographically patterned quartz substrate (gray), supported membrane (green), and upper (red) membrane in the experiment of Parthasarathy et al. [54] (reproduced with permission from [54]).

Consider a biomembrane composed of two components, for example, two different lipid molecules or two different lipid phases, that lies on a smooth nonflat surface (in their experiment, Parthasarathy et al. [54] used a double membrane system to decouple the main membrane from the underlying substrate: a “supporting” membrane of uniform composition was deposited on the substrate in order to chemically decouple the “main” membrane from the substrate, and only then the “main” membrane was introduced on top of it) that has a geometry (shape) of a continuously alternating curvature with one-dimensional periodicity; see Figure 1(b). The free energy of the biomembrane takes the form [36]

(1) F=∫A12kHϕH−H0ϕ2+fϕ+12γ∇ϕ2dA,

where integration is performed over the entire surface area of the membrane, *A*. Above, the first term is the (Helfrich) bending energy [25, 55] which depends on the mean curvature, *H*, the second term, *f*, is the specific mixing energy, and the last term describes the energetic penalty for spatial composition gradients. In addition, *ϕ* : *A* → [0,1] describes the mole fraction of the second component, which we also refer to as local composition or concentration. A few comments are in order: (i) functional (1) does not include a stretching energy term or a Gauss-curvature bending energy term. The reason is that the biomembrane lies freely on a smooth surface; thus its stretching energy vanishes. Also, the Gauss-curvature bending energy vanishes everywhere since the imposed geometry has a 1D periodicity, which results in one of the two principal curvatures being zero. (ii) The two components of the biomembrane differ in their mechanical properties; namely, they have different bending stiffness, *k*_*H*_, and spontaneous curvature, *H*_0_. Thus, inhomogeneity induces local spontaneous curvature and stiffness that depend on local composition, *ϕ*. (iii) The specific mixing energy, *f*, combines the aggregation enthalpy and the entropy of mixing. A simple model that is often adopted for modeling the mixing energy is the so-called “gas lattice” or “regular solution” model, which takes the form [36, 42]

(2) fϕ=kBT0ρ0T−ϕln⁡ϕ+1−ϕln⁡1−ϕ+2ϕ1−ϕ.

Here, *k*_*B*_ is the Boltzmann's constant, *T*_0_ is the critical temperature defined as the temperature at which the mixing energy changes from single-well to double-well structure, *ρ*_0_ is the lipid density (number of molecules per unit area), and $\overset{-}{T}=T/T_{0}$ is the nondimensional temperature. Consequently, *f* is convex (miscible) at temperatures $\overset{-}{T}>1$ but nonconvex with double-well structure at temperatures $\overset{-}{T}<1$.

It is convenient to define *g*(*ϕ*) = (1/2)*k*_*H*_(*ϕ*)(*H* − *H*_0_(*ϕ*))^2^ + *f*(*ϕ*); that is,

(3) $$\begin{array}{c} F=\overset{}{\underset{A}{\int }}\left(\;g\left(\;\phi \;\right)+\frac{1}{2}\gamma {\left|\;\nabla \phi \;\right|}^{2}\;\right)dA, \end{array}$$

The chemical potential, *μ*, reflects the change in free energy due to a small change in local concentration. In our case, the concentration is a spatial field; thus the chemical potential is a spatial field as well. Accordingly, the chemical potential is defined in terms of the (functional) variation of *F* with respect to the concentration field [36]; that is,

(4) δF=∫μδϕ dA⟹μ=g,ϕ−γ∇2ϕ,

where ( )_,*ϕ*_ denotes partial differentiation with respect to *ϕ*.

Equilibrium configurations correspond to local minima of the free energy, subjected to the relevant constraints. In our case, the system is closed so the total number of molecules of each type is preserved:

(5) $$\begin{array}{c} \overset{}{\underset{A}{\int }}\phi \;dA=\text{const.} \end{array}$$

In order to calculate equilibrium configurations, one can use the method of Lagrange multipliers and introduce the functional

(6) $$\begin{array}{c} F^{*}=F-\lambda_{L}\int \phi \;dA. \end{array}$$

An equilibrium configuration must satisfy the condition *δF*^*∗*^ = 0; that is,

(7) $$\begin{array}{c} \mu =\lambda_{L}=\text{const.} \end{array}$$

This result implies that, at equilibrium, the chemical potential field must be uniform. Note that the value of this potential is not a priori known (it needs to be calculated) and depends on the specific parameters of the problem at hand, such as the overall (average) concentration and surface geometry.

In what follows, we formulate the equations that govern the evolution of the concentration field *ϕ*. These equations assume that *ϕ* evolves such that the free energy decreases at the highest rate. In numerical analysis, this is often termed the “steepest descent” method. Based on this approach, equilibrium configurations can be found by solving these equations (calculating *ϕ*) for long times. In reality, the details of the time-dependent evolution of the composition field *ϕ* may not follow exactly this strategy. Nevertheless, our main interest is in the final (equilibrium) configuration rather than in the details of the evolution, and we consider this numerical scheme as a reasonable evolution strategy. The composition, *ϕ*, varies as long as the chemical potential field is nonuniform, or, in other words, gradients exist. Flow is from high potential to low potential; thus the concentration flux, **J**, takes the form of a generalized Fick's law:

(8) $$\begin{array}{c} J=-M\nabla \mu , \end{array}$$

where *M* > 0 is the mobility and vectors are identified by bold-face font. Since the overall number of molecules in the biomembrane does not change during the time scale of the experiment, changes in the concentration field are attributed only to flux. Hence, we write the following conservation law:

(9) $$\begin{array}{c} \phi_{,t}+\nabla \cdot J=0. \end{array}$$

Plugging (9) into (8) we conclude with the governing equation for the evolution of *ϕ*:

(10) $$\begin{array}{c} \phi_{,t}=M\Delta \mu =M\Delta \left(\;g_{,\phi }-\gamma \Delta \phi \;\right), \end{array}$$

with

(11) g,ϕ=f,ϕ+12kH,ϕH−H02+kHH−H0H0,ϕ,

(12) f,ϕ=kBT0ρ0T−ln⁡ϕ1−ϕ+2−4ϕ.

### 2.2. Nondimensional Analysis

Next, we rewrite the governing equation in a nondimensional form. Besides the convenient formulation, this procedure enables us to identify of the nondimensional quantities that govern the behavior. To this end, we consider the characteristic scales of energy, length, and time.

The coefficient of the mixing energy *k*_*B*_*T*_0_*ρ*_0_ sets the typical (specific, per unit area) energy scale. By dividing (11) with the energy scale, *k*_*B*_*T*_0_*ρ*_0_, we obtain the nondimensional form of *g*_,*ϕ*_:

(13) g−,ϕg,ϕkBT0ρ0=T−ln⁡ϕ1−ϕ+2+4ϕ︸f−,ϕ+12kH,ϕH−H02kBT0ρ0︸II+kHH−H0H0,ϕkBT0ρ0︸III.

By introducing the typical bending stiffness, *k*_*H*_^*∗*^, and a typical mean curvature of the surface, *H*^*∗*^, the second term in (13) reads

(14) 12kH,ϕH−H02kBT0ρ0=mbH−−H−02;mb=12H∗2kH∗kBT0ρ0k−H,ϕ,

where $\overset{-}{H}=H/H^{*}$ and ${\overset{-}{k}}_{H}=k_{H}/k_{H}^{*}$ denote nondimensional curvature and bending stiffness, respectively, and *H*^*∗*^ is taken as the maximal curvature of the surface. The bending stiffness of each of the two membrane components (separated phases) is denoted by *k*_*H*_|_*ϕ*=0_ = *k*_*I*_ and *k*_*H*_|_*ϕ*=1_ = *k*_*II*_. For specificity, and without loss of generality, we approximate the dependence of *k*_*H*_ with *ϕ* by a linear relation [43]. Thus, *k*_*H*,*ϕ*_ = *k*_*II*_ − *k*_*I*_ and we choose *k*_*H*_^*∗*^ = *k*_*I*_. Similarly, we approximate the dependence of *H*_0_ with *ϕ* by a linear relation, that is, *H*_0,*ϕ*_ = *H*_*II*_ − *H*_*I*_. Plugging these relations into (14) we have that

(15) $$\begin{array}{c} m_{b}=\frac{1}{2}\frac{k_{I}{\left(H^{*}\right)}^{2}}{k_{B}T_{0}\rho_{0}}\left(\;{\overset{-}{k}}_{II}-{\overset{-}{k}}_{I}\;\right). \end{array}$$

The nondimensional quantity *m*_*b*_ represents the ratio between the typical bending energy and the mixing energy and also reflects the contribution of the differences between the bending stiffness of the two components.

Applying a similar procedure to the third term in (13), we find that

(16) $$\begin{array}{c} \frac{k_{H}\left(H-H_{0}\right){H_{0}}_{,\phi }}{k_{B}T_{0}\rho_{0}}=m_{h}{\overset{-}{k}}_{H}\left(\;\overset{-}{H}-{\overset{-}{H}}_{0}\;\right), \end{array}$$

where

(17) $$\begin{array}{c} m_{h}=\frac{k_{I}{\left(H^{*}\right)}^{2}}{k_{B}T_{0}\rho_{0}}\cdot \left(\;{\overset{-}{H}}_{II}-{\overset{-}{H}}_{I}\;\right). \end{array}$$

The nondimensional quantity *m*_*h*_ represents the ratio between the bending energy and the mixing energy, similarly to *m*_*b*_; however, it reflects the contribution of the differences in spontaneous curvatures (rather than bending stiffness) between the two phases.

Nondimensional spatial coordinates (location), ${\overset{-}{x}}_{i}=x_{i}/\lambda \;\;(i=1,2)$, are defined using the length of the 1D period of the surface curvature, *λ*. The corresponding nondimensional gradient operator is $\overset{-}{\nabla }=\lambda \nabla$. Similarly, by introducing the characteristic time scale, *τ* = *λ*^2^/*Mk*_*B*_*T*_0_*ρ*_0_, we have that $\overset{-}{t}=t/\tau$ and $\partial /\partial \overset{-}{t}=\tau \partial /\partial t$. Using these definitions along with relations (13)–(17), we conclude with the nondimensional form of the governing equation (10):

(18) $$\begin{array}{c} \frac{\partial \phi }{\partial \overset{-}{t}}=\overset{-}{\Delta }\left(\;{\overset{-}{g}}_{,\phi }-m_{1}\cdot \overset{-}{\Delta }\phi \;\right), \end{array}$$

where

(19) g−,ϕ=T−ln⁡ϕ1−ϕ+2−4ϕ+mbH−−H−02+mhK−H−−H−0,

(20) m1=1λ2γkBT0ρ0.

Equation (18) and thus the behavior of the biomembrane are governed by three nondimensional quantities; these are *m*_*b*_, *m*_*h*_, and *m*_1_ defined in (15), (17), and (20), respectively.

## 3. Numerical Results

In this section, we present numerical results focusing on the influence of the nondimensional quantities that were identified in the previous section on the response of a biomembrane with imposed geometry.

### 3.1. Numerical Scheme and Additional Considerations

**Figure 2:**
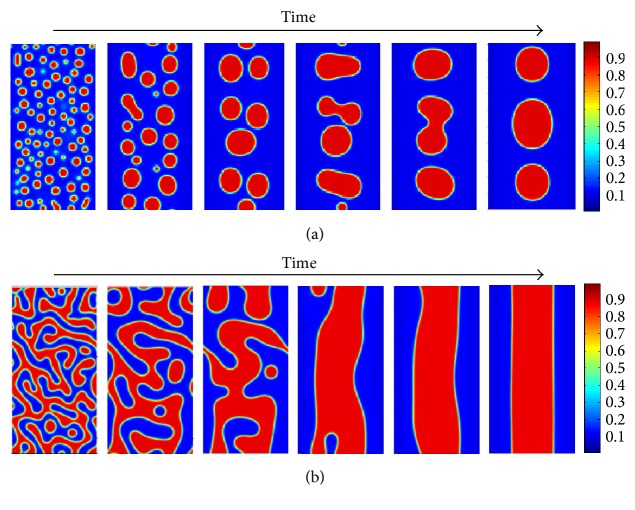
Snapshots of typical simulations. (a) *m* = 2, *m*_*b*_ = 0.1, *m*_*h*_ = 0, and *ϕ*_average_ = 0.3 at times $\overset{-}{t}=\left(1,10,30,50,60,80\right)\cdot 10^{4}$ (from left to right). The simulation was stopped at $\overset{-}{t}=5\cdot 10^{6}$ with no visible change compared to the last snapshot shown here. (b) Same as (a), but with *ϕ*_average_ = 0.5, which is in the spinodal region of *f*(*ϕ*). Videos of these simulations are provided in Supplementary Material available online at https://doi.org/10.1155/2017/7275131.

In our numerical simulations we calculate the evolution in time of the composition field, *ϕ*, in a rectangular portion of the surface. The dimensions of this rectangle are *λ* × 2*λ*, as illustrated in Figure 1, subjected to periodic boundary conditions. This choice of domain size enables the description of the occurrences while significantly reducing computational effort (compared to simulating the entire biomembrane). The rectangular domain is discretized into *n*_1_ points in the horizontal (*x*_1_) direction and *n*_2_ points in the *x*_2_-direction, with equal spacing in both directions, that is, Δ*x*_1_ = Δ*x*_2_ = 1/(*n*_1_ − 1). Typically, a value of *n*_1_ = 100 was used to discretize a single length unit (or *λ* in dimensional length). We adopt a conservative second-order numerical scheme with adaptive time stepping [44, 45, 56, 57].  Figure 2 shows snapshots of the composition field at different times, where a color scale is used to describe the level of local concentration.

All simulations start from random noncorrelated values near *ϕ*_average_, which defines the overall (average) composition. These small (less than 0.01*ϕ*_average_) random deviations from the average composition are necessary, in some of the cases, in order to break the “symmetry” and allow for initiation of the evolution. In most simulations we have used *ϕ*_average_ = 0.3 in accordance with the experiment of Parthasarathy et al. [54]. Still, we have also studied the influence of overall composition on the phase behavior.

In the experiments of Parthasarathy et al. [54], the curvature field was calculated from AFM measurements of the substrate height profile. This imposed geometry (curvature) can be well captured by the simple functional relation

(21) $$\begin{array}{c} \overset{-}{H}\left(\;{\overset{-}{x}}_{1},{\overset{-}{x}}_{2}\;\right)=\frac{{\left({\overset{-}{x}}_{1}-0.5\right)}^{m}}{0.5^{m}}. \end{array}$$

Here, ${\overset{-}{x}}_{1}\in [0,1]$ is the nondimensional coordinate, where the geometry of the surface has a period of one. The coefficient *m* governs the “shape” of the curvature field; higher *m* values are associated with a larger region, around ${\overset{-}{x}}_{1}=0.5$, having flat geometry. A value of *m* = 2 is typical to the surface profile used in the experiments of Parthasarathy et al. [54].

The main purpose of the numerical investigation is to study the interplay between the imposed geometry (curvature) and composition of the biomembrane. Hence, we focus our attention on the influence of the nondimensional quantities *m*_*b*_, *m*_*h*_, and *m* on the final (steady state) composition field, with dimensional values of *λ* = 2 *μ*m, *T*_0_ ~ 300 K, *ρ*_0_ ~ 5 · 10^4^ [*μ*m^−2^], and *γ* = 10^−19^ [J] [36, 54] implying that *m*_1_ ~ 10^−4^. The quantities *m*_*b*_ and *m*_*h*_ represent the intensity of coupling between curvature and composition through the difference between the bending stiffness and the spontaneous curvatures of the two phases, respectively. Different values of *m*, on the other hand, are associated with surfaces having the same maximum curvature but different topography. In the experiments of Parthasarathy et al. [54], the authors intentionally chose a lipid bilayer with zero spontaneous curvature. Thus, we consider first this case, namely,

(22) H0ϕ=0⟹g−,ϕ=T−ln⁡ϕ1−ϕ+2−4ϕ+mbH−2.

### 3.2. The Effect of *m*_*b*_

Motivated by the experiments of Parthasarathy et al. [54], we consider first the case of zero spontaneous curvature (22), aiming at reproducing the experimental observations regarding the influence of the surface topography on the phase behavior. In particular, it was suggested that there exists a critical curvature above which the composition morphology is strongly correlated with the surface geometry. Below this critical curvature, the position of domains is rather random and does not seem to register with the geometry of the surface.

In our model, the magnitude of the surface curvature is accounted for by the nondimensional quantity *m*_*b*_ (15), where larger values of *m*_*b*_ represent higher surface curvature. Note that the mathematical structure of *m*_*b*_ indicates that the influence of the surface curvature, through *H*^*∗*^^2^, is equivalent to that of the difference between the stiffness of the two phases, $k_{I}\left({\overset{-}{k}}_{II}-{\overset{-}{k}}_{I}\right)$. Thus a higher value of *m*_*b*_ reflects either higher curvature of the imposed geometry or a biomembrane with higher stiffness (or a combination of the two). In the experiments of Parthasarathy et al. [54], the response of biomembranes with similar composition was studied by subjecting them to surfaces with different curvatures. Thus, in these experiments *m*_*b*_ was altered by changing the surface feature *H*^*∗*^.

**Figure 3:**
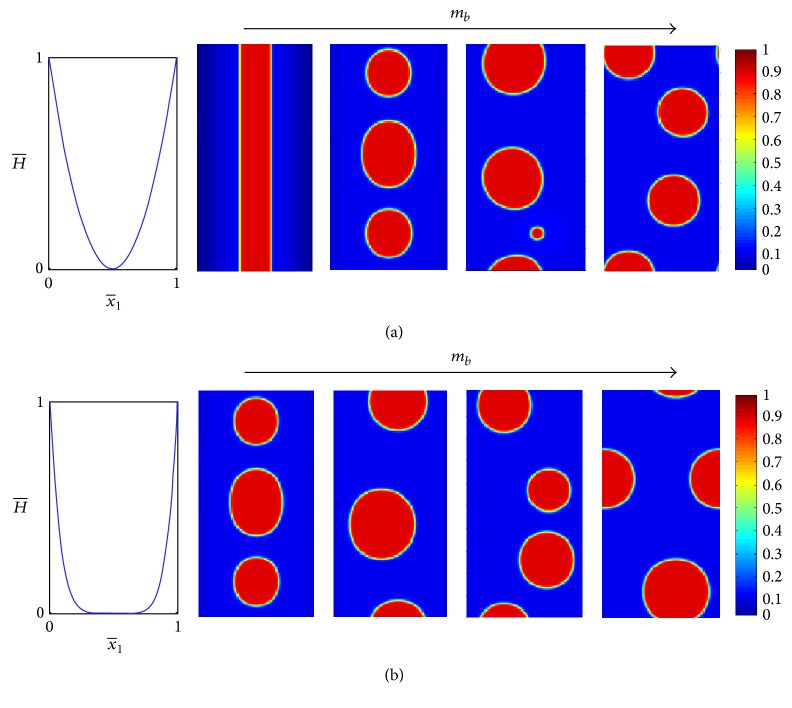
Final (steady state) composition field for different values of *m*_*b*_ and *H*_0_(*ϕ*) = 0, for (a) *m* = 2 and (b) *m* = 6. In both (a) and (b), the leftmost plots show the imposed curvature, while the other four plots illustrate, from left to right, the composition field $\phi ({\overset{-}{x}}_{1},{\overset{-}{x}}_{2})$ for *m*_*b*_ = 1, 10^−1^, 10^−3^, 10^−4^. *ϕ*_average_ = 0.3.

Figure 3 shows the long-time (steady state) solution of the composition field obtained from numerical simulations for various values of *m*_*b*_. These results demonstrate that *m*_*b*_ has a significant influence on the ordering of the two phases as a result of the reciprocity between bending stiffness and surface topography. In particular, energy considerations favor configurations where the stiffer phase is located at regions of lower curvature. From these images, we can learn more on the effect of *m*_*b*_ on the biomembrane behavior. As *m*_*b*_ decreases, the domains become less organized and show lower correspondence with the surface topography. For example, with *m* = 2 and *m*_*b*_ = 10^−1^, the segregated domains of the stiffer (red) phase are oval and perfectly centered at ${\overset{-}{x}}_{1}=0.5$, while for *m*_*b*_ = 10^−4^ the location of the domains is random with no particular preference, and their shape is almost perfectly round due to surface tension which becomes more dominant for small values of *m*_*b*_. These results suggest that the surface geometry affects the phase behavior only when the surface curvature is high enough. In accordance with observations of Parthasarathy et al. [54], values of *m*_*b*_≃10^−4^ or smaller correspond to negligible influence of the imposed geometry, while increasingly higher values of *m*_*b*_ result in an increasing effect.

Recall that *m*_*b*_ also reflects the difference between the bending stiffness of the phases. Hence, we generalize the conclusion of Parthasarathy et al. [54], which was specific to the lipid phases used in their experiments. By understanding the dual role of *m*_*b*_, we conclude that higher stiffness ratios between the two phases decrease the magnitude of the minimal surface features required to couple between the surface topography and the biomembrane composition. This conclusion is somewhat intuitive, but now the mathematical structure of *m*_*b*_ describes it quantitatively.

The parameter *m* governs the width of the flat section of the surface topography. Its influence on the phase behavior is exemplified by comparing the results of Figures 3(a) and 3(b). Due to the energy-related reasoning discussed above, surfaces with smaller values of *m* constrict the domains of the stiffer phase to a narrower region in the middle of the surface (where the curvature is small). As a result, the circular shape of the domains becomes more oval as *m* decreases (or *m*_*b*_ increases), until the point where constriction is so tight that all domains merge and form a single strip; see for example, Figure 3(a) with *m* = 2 and *m*_*b*_ = 1. Note that such extreme morphology has not been observed in the experiments. The reason is that this type of behavior requires a combination of small *m* and very large *m*_*b*_. Specifically, the maximum values reached in the experiments of Parthasarathy et al. [54] were *m*_*b*_≃0.01 with *m* = 2.

**Figure 4:**
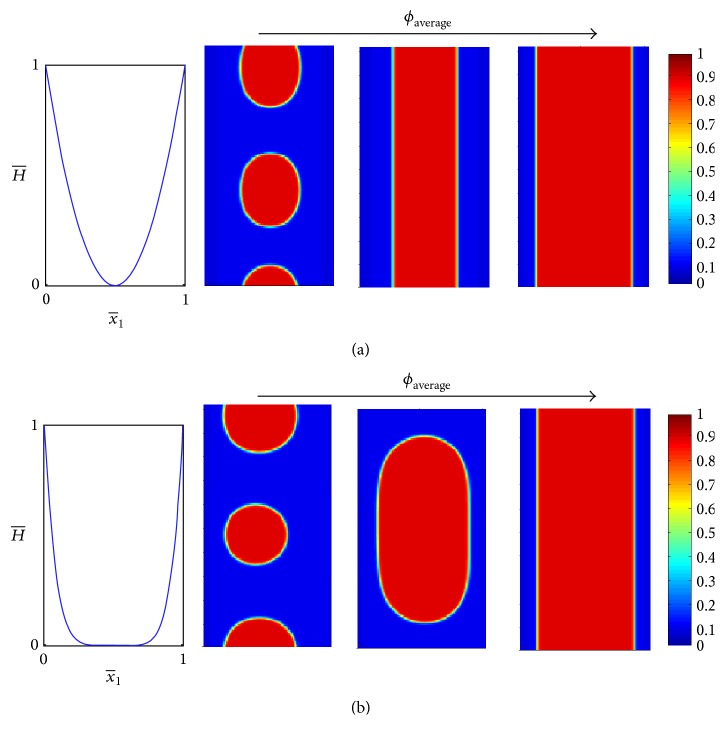
The effect of overall (average) composition, *ϕ*_average_, on the final (steady state) composition field. (a) *m* = 2 and (b) *m* = 6. In both (a) and (b), the leftmost plots show the imposed curvature, while the other four plots illustrate, from left to right, the composition field $\phi ({\overset{-}{x}}_{1},{\overset{-}{x}}_{2})$ for *ϕ*_average_ = 0.3, 0.5, 0.7. *m*_*b*_ = 0.1 and *H*_0_(*ϕ*) = 0.

*Overall Concentration*. The stripe morphology can take place for lower values of *m*_*b*_ by increasing the overall (average) concentration *ϕ*_average_. This is exemplified in Figure 4, which shows the effect of overall composition on the ordering of the two phases. For example, with *m* = 2 and *m*_*b*_ = 0.1 the stripe morphology does not appear with overall concentration of *ϕ*_average_ = 0.3 but appears with *ϕ*_average_ = 0.5 or higher. Also, with *m* = 6 and *ϕ*_average_ = 0.3, the stripe morphology does not appear even for *m*_*b*_ = 1; see Figure 3(b) but does appear at *m*_*b*_ = 0.1 with *ϕ*_average_ = 0.7; see Figure 4(b). The reasoning for this phenomenon is that the (energetic) avoidance of the stiffer phase from high curvature constricts it to a “stripe” of relatively small curvature in the middle. If the overall concentration of the stiffer phase is low, it forms small round domains that remain separated since they are far enough apart within this stripe. On the other hand, if the overall concentration of the stiffer phase is higher, the stiffer phase almost fills the stripe, which leads to the formation of oval domains, for example, Figure 4(b) with *ϕ*_average_ = 0.5. At even higher concentrations, all domains merge into a single strap in order to minimize line (surface) tension energy, as shown in Figure 4(b) with *ϕ*_average_ = 0.7.

Similarly to Figure 3, the difference between Figures 4(a) and 4(b) stems from the competition between the bending energy and the line tension energy. When the surface has a wider flat section (and the overall concentration is small), the bending energy is less significant within this flat section and the system reduces the line tension energy by forming circular domains that have shorter phase boundaries. However, when the surface has a relatively narrow flat section, the system must restrict the stiffer phase to a narrow strap in order to reduce the bending energy, which becomes significant outside the strap, on the expense of the energy penalty for longer phase boundaries. This leads to the formation of oval domains, and, in cases where the overall concentration is high enough, it leads to the stripe morphology. 

**Figure 5:**
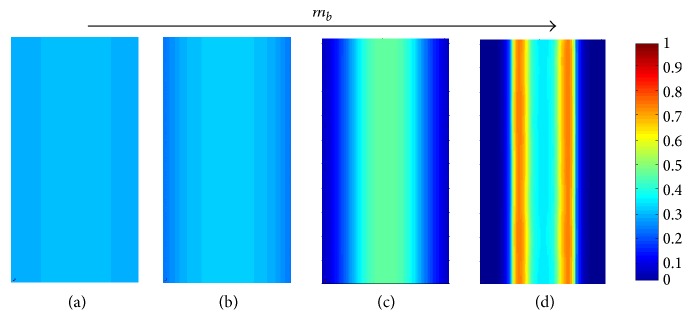
Behavior above critical temperature. (a) *m*_*b*_ = 0.01, (b) *m*_*b*_ = 0.1, (c) *m*_*b*_ = 1, (d) *m*_*b*_ = 10. *m* = 2, and *ϕ*_average_ = 0.3.

*Behavior above the Critical Temperature*. The simulations presented above, just like the experiments of Parthasarathy et al. [54], were performed at a temperature lower than the miscibility temperature *T*_0_. Hence, the double-well structure of the interaction energy, *f*(*ϕ*), drives the system towards phase separation and formation of domains. In turn, the topography of the surface (the imposed geometry) affects the spatial ordering and shape of these domains. On the other hand, above the critical temperature, the interaction energy favors miscibility, that is, uniform composition. Such behavior was indeed observed in the experiments as well as in our numerical simulations. Nevertheless, our simulations also show that in cases where the magnitude of the surface features (curvature) is high enough, the imposed geometry can lead to the formation of a spatially nonuniform composition field and to geometry-induced phase separation above the critical temperature; see Figure 5. One must note that this type of nonstandard behavior requires high values of *m*_*b*_. Also, unlike standard phase separation that often exhibits round domains, here the spatial distribution of the composition field is dominated almost completely by the surface features which gives rise only to a stripes-like ordering. Unfortunately, the surface features used in the experiment of Parthasarathy et al. [54] were much smaller; thus validation of this phenomenon still awaits experimental confirmation.

### 3.3. The Role of Spontaneous Curvature

A comparison between the definitions of *m*_*b*_ (15) and *m*_*h*_ (17) suggests that the difference in spontaneous curvatures, ${\overset{-}{H}}_{I}$ and ${\overset{-}{H}}_{II}$, and the difference in bending stiffness, ${\overset{-}{k}}_{I}$ and ${\overset{-}{k}}_{II}$, of the two phases have a similar influence on the biomembrane composition. In particular, (13) indicates that bending energy drives the stiffer phase towards smaller surface curvatures (second term in (13)) and also the phase with higher spontaneous curvature towards locations of higher surface curvature (third term in (13)). Hence, the differences in the bending stiffness and in spontaneous curvature between the two phases make each of the two phases favor (energetically) different locations on the surface. The magnitude of this effect is largely associated with the magnitude of the nondimensional quantities *m*_*b*_ and *m*_*h*_. These two quantities have a similar mathematical structure which expresses the relative importance of the bending energy compared to the interaction energy multiplied by the difference in bending stiffness or spontaneous curvatures of the two phases, respectively. Hence, the role of *m*_*h*_ and its influence on the biomembrane response when subjected to imposed geometry seems to be qualitatively similar to that of *m*_*b*_, which has been studied in the previous section. Nevertheless, we note a fundamental difference between the second and third terms in (13). That is, while *m*_*b*_ multiplies ${\left(\overset{-}{H}-{\overset{-}{H}}_{0}\right)}^{2}$, *m*_*h*_ multiplies $\left(\overset{-}{H}-{\overset{-}{H}}_{0}\right)$. The importance of this difference is twofold: (i) changes in the geometry of the biomembrane, for example, by subjecting it to a surface with higher curvature, affect more significantly the second term compared to the third term in (13). In particular, in the case where *m*_*b*_ and *m*_*h*_ are comparable, the third term in (13) is more sensitive to the imposed geometry. Thus, roughly speaking, the role of the bending stiffness in ordering the biomembrane composition is more significant than that of the spontaneous curvature. (ii) The sign of the third term in (13) depends on the sign of *H* − *H*_0_, while that of the second term does not.

**Figure 6:**
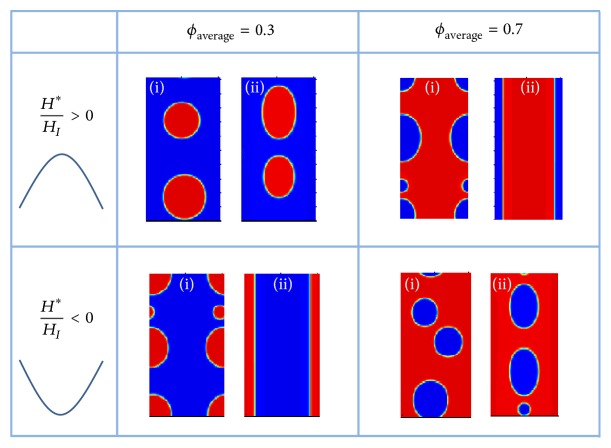
The effect of spontaneous curvature for different values of *ϕ*_average_ and sign of the surface curvature. Each plot shows the final (steady state) spatial distribution of the composition field *ϕ*. In each of the four “windows” the left and right plots, (i) and (ii), correspond to ${\overset{-}{H}}_{I}=0.1$ and ${\overset{-}{H}}_{I}=1$, respectively. *m* = 2, *k*_*I*_ = *k*_*II*_, and *H*_*II*_ = −*H*_*I*_.

Following the discussion above, we focus our attention below on demonstrating the consequences of the fact that the sign of the third term in (13) depends on the sign of *H* − *H*_0_. To this end, we consider the simple case where *k*_*II*_ = *k*_*I*_ and *H*_*II*_ = −*H*_*I*_ are together with the typical dimensional values of *k*_*I*_ ~ 10^−19^ J, |*H*^*∗*^| ~ 1 *μ*m^−1^, *T*_0_ ~ 300 K, and *ρ*_0_ ~ 5 · 10^4^ [*μ*m^−2^] [36, 54], which imply that *m*_*b*_ = 0, *H*_0_ = (1 − 2*ϕ*)*H*_*I*_, and $m_{h}\sim -10^{-3}{\overset{-}{H}}_{I}$.  Figure 6 shows the difference in the final (steady state) spatial distribution of the biomembrane composition, *ϕ*, when imposed on convex and concave surfaces and for different values of ${\overset{-}{H}}_{I}$ and of the average concentration. As expected, higher values of *H*_*I*_ (which also mean higher difference between the spontaneous curvatures of the two phases) increase the correlation between the observed composition patterns and the surface topography. High values of *H*_*I*_ lead to the formation of oval domains and when *H*_*I*_ is high enough to the stripe morphology. These features are qualitatively similar to the behavior observed in the previous section where the effect of the difference in bending stiffness was studied. A fundamental difference, however, is in the distribution of the biomembrane composition, *ϕ*, when imposed to convex or concave surfaces. This is illustrated by comparing results in Figure 6 that differ only in the sign of *H*^*∗*^ (top row compared to the bottom row). A few comments are in order: (i) the different sign of the surface curvature inverts the spatial location favored by each phase. Importantly this is not the case when the two phases differ only in their bending stiffness; (ii) the inversion of preferred locations leads to a different constriction exhibited by the phases; this may result in significant changes in the observed morphology; for example, oval compared to circular domains or stripe morphology compared to oval domains; (iii) the specific example studied here, with *H*_*II*_ = −*H*_*I*_, is antisymmetric with respect to (sign of) the surface curvature. Thus, cases involving opposite sign of the surface curvature combined with “opposite” average concentrations are expected to yield opposite spatial organization, that is, similar morphology with inverted phase locations. This feature is indeed observed when comparing results in the upper-left and bottom-right “windows” of Figure 6, as well as upper-right and bottom-left “windows” in the same figure.

## 4. Summary and Conclusions

We formulated a simple mathematical model to study the spatial ordering of composition in a two-component biomembrane that is subjected to prescribed (imposed) geometry. The mathematical model does not account for possible anisotropy of the membrane constituents or for possible interaction between lipids in the two leaflets of the bilayer membrane. In addition, the numerical scheme, which follows the steepest descent method, leads to metastable equilibrium configurations associated with local minima of the free energy. We note, however, that applying the numerical scheme to slightly different initial conditions or to a larger domain of solution did not change the essence (topology) of the solution. Based on this model, we identified key nondimensional quantities that govern the biomembrane response and performed numerical simulations to quantitatively study their influence. Our numerical results show that the geometry-driven ordering of the biomembrane composition is largely governed by the difference between the nondimensional bending stiffness and spontaneous curvatures of each phase, while the magnitude of this phenomenon is proportional to the ratio between the bending energy and the (chemical) interaction energy of the phases. Roughly speaking, energy considerations favor configurations in which the phase that is stiffer and has smaller spontaneous curvature is located at regions having smaller curvature. The numerical simulations reproduced the experimental observation of Parthasarathy et al. [54], who found that above a critical surface curvature the composition morphology is strongly correlated with the surface geometry, while below this threshold, the position of domains is rather random and does not register with the geometry of the surface. Careful investigation of our model equations enable us to generalize this experimental observation beyond the specific lipid phases used in that experiment.

An important advantage of a mathematical model is that it enables studying the behavior at various settings with minimal effort and resources, before entering the lab. The agreement of our model results with experimental observations strengthens our confidence in the model and numerical scheme and opens the door to examining new and different conditions than those used in the original experiments. For example, we have demonstrated that, if the surface geometry is properly designed, phase separation can occur above the critical temperature. Such curvature-induced phase separation above the critical temperature awaits experimental examination. Also, we propose a systematic procedure to determine which mechanism, the difference in bending stiffness or difference in spontaneous curvatures of the two phases, dominates the coupling between shape and composition. The procedure is based on the observation that the mechanism associated with the difference in bending stiffness depends on the magnitude of the surface curvature but indifferent to the sign (direction) of the curvature. On the contrary, the mechanism related to the spontaneous curvatures strongly depends on both magnitude and sign (direction) of the surface curvature. The consequences of these differences have been demonstrated by a set of simulations.

## Supplementary Material

Movies showing the evolution in time for the simulations shown in Figure 2.
